# TiO_2_ Photocatalyzed Oxidation of Drugs Studied by Laser Ablation Electrospray Ionization Mass Spectrometry

**DOI:** 10.1007/s13361-018-2120-x

**Published:** 2019-01-07

**Authors:** Fred A. M. G. van Geenen, Maurice C. R. Franssen, Ville Miikkulainen, Mikko Ritala, Han Zuilhof, Risto Kostiainen, Michel W. F. Nielen

**Affiliations:** 1 Laboratory of Organic Chemistry, Wageningen University, Stippeneng 4, 6708 WE Wageningen, The Netherlands; 2 TI-COAST, Science Park 904, 1098 XH Amsterdam, The Netherlands; 3 Department of Chemistry, University of Helsinki, P.O. Box 55, 00014 Helsinki, Finland; 4 School of Pharmaceutical Sciences and Technology, Tianjin University, 92 Weijin Road, Tianjin, People’s Republic of China; 5 Department of Chemical and Materials Engineering, King Abdulaziz University, Jeddah, Saudi Arabia; 6 Drug Research Program, Division of Pharmaceutical Chemistry and Technology, Faculty of Pharmacy, University of Helsinki, P.O. Box 56, 00014 Helsinki, Finland; 7 RIKILT, Wageningen University & Research, P.O. Box 230, 6700 AE Wageningen, The Netherlands

**Keywords:** Laser ablation, Mass spectrometry, Oxidation, Photocatalysis, Ambient ionization, Titanium dioxide, Selective androgen receptor modulator

## Abstract

In drug discovery, it is important to identify phase I metabolic modifications as early as possible to screen for inactivation of drugs and/or activation of prodrugs. As the major class of reactions in phase I metabolism is oxidation reactions, oxidation of drugs with TiO_2_ photocatalysis can be used as a simple non-biological method to initially eliminate (pro)drug candidates with an undesired phase I oxidation metabolism. Analysis of reaction products is commonly achieved with mass spectrometry coupled to chromatography. However, sample throughput can be substantially increased by eliminating pretreatment steps and exploiting the potential of ambient ionization mass spectrometry (MS). Furthermore, online monitoring of reactions in a time-resolved way would identify sequential modification steps. Here, we introduce a novel (time-resolved) TiO_2_-photocatalysis laser ablation electrospray ionization (LAESI) MS method for the analysis of drug candidates. This method was proven to be compatible with both TiO_2_-coated glass slides as well as solutions containing suspended TiO_2_ nanoparticles, and the results were in excellent agreement with studies on biological oxidation of verapamil, buspirone, testosterone, andarine, and ostarine. Finally, a time-resolved LAESI MS setup was developed and initial results for verapamil showed excellent analytical stability for online photocatalyzed oxidation reactions within the set-up up to at least 1 h.

**Electronic supplementary material:**

The online version of this article (10.1007/s13361-018-2120-x) contains supplementary material, which is available to authorized users.

**Figure:**
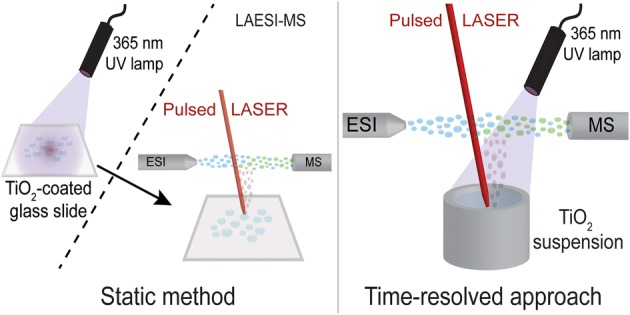
Graphical Abstract

## Introduction

Metabolism of drugs is an important aspect of their efficacy after administration to the body. One aspect of human metabolism is the excretion of xenobiotics—e.g., drugs or carcinogens—by biochemical transformations. In pharmacology, these transformations can also be exploited for the activation of prodrugs. The metabolism of drugs is often divided into two phases, biotransformation and bioconjugation, respectively. The biotransformation, phase I, plays an important role in the deactivation of drugs and activation of prodrugs and comprises reactions such as oxidation, reduction, and hydrolysis. In drug discovery, it is important to identify these modifications as early as possible to screen for possible pharmacologically active compounds [1, 2]. Commonly, the metabolism of drug candidates is evaluated by in vitro biotransformations (with microsomes or recombinant enzymes), which are time-consuming and relatively expensive [2]. Since the major class of reactions in phase I metabolism are oxidation reactions [3], also non-enzymatic methods could be used for rapid phase I reactions to eliminate drug candidates with an undesired metabolism. Apart from electrochemistry approaches [4, 5], titanium dioxide (TiO_2_) photocatalysis is a simple non-enzymatic method to generate oxidation reaction products similar to those obtained by phase I biochemical transformations [6–13].

In TiO_2_ photocatalysis (see refs [14, 15] for reviews), TiO_2_ is used as a material that is able to catalyze oxidation and reduction reactions when exposed to ultra-violet (UV) radiation [16–18]. The absorbance of a photon by TiO_2_ with an energy greater than its bandgap (3.2 eV, 385 nm) will excite a valence electron, producing a free electron in the conduction band and leaving a hole in the valence band. Both the hole and free electron can react with other molecules that are present at the material surface, like molecular oxygen and water molecules. Molecular oxygen can be reduced to superoxide (O_2_^−^), which is a very strong oxidizing agent of organic molecules [19]. In both the oxidation (by photogenerated holes) and reduction (by the free electrons) of water molecules, hydroxyl radicals are produced. Hydroxyl radicals are highly oxidative and have been used extensively as a tool for oxidation reactions with drug candidates [6–13].

Mass spectrometry (MS), often coupled with liquid chromatography (LC), is a well-established analysis technique for the identification and quantification of drugs and their metabolites following in vitro and in vivo studies. Hence, LC-MS has been used as analytical method of choice for identification of the drug reaction products in most TiO_2_ photocatalyzed oxidation studies [6–12]. This method requires the removal of TiO_2_ particles prior to LC-MS analysis, introducing pretreatment steps and thus limiting sample throughput. Improvements have been achieved with a nanoreactor electrospray ionization chip (TiO_2_-μPESI) containing integrated TiO_2_-coated micropillars for direct MS analysis [13]. Apart from this approach, a simple TiO_2_-coated rotating platform was recently introduced in combination with desorption electrospray ionization (DESI) MS, providing the opportunity to increase throughput of oxidation reactions up to four samples per minute [20]. Although these MS methods do not allow for the separation of isomeric reaction products, MS/MS provides information on the modification site. A drawback in current TiO_2_ photocatalysis systems is the possibility of continuous oxidation of formed reaction products, as often multiple, consecutive oxidation reactions might take place. This makes it difficult to identify sequential oxidation steps. An online, time-resolved based method would therefore be a real asset for identification of products, and to follow consecutive and/or parallel modifications in real time.

Laser ablation electrospray ionization (LAESI) is an ambient MS technique used in the analysis of, e.g., tissues, food contaminants, solid materials, and liquids [21–29]. It uses a 2.94 μm mid-infrared pulsed laser that addresses hydroxyl moieties leading to the efficient ablation of sample material that is subsequently extracted by charged electrospray droplets upon MS analysis. LAESI has previously been demonstrated its ability to allow monitoring of enzymatic reactions in time, directly from a well plate [28]. In a LAESI TiO_2_ photocatalysis MS system, reaction products in the ablation plume would simply be extracted by electrospray droplets for MS detection, without any need for removal of TiO_2_ particles. Temporal resolution would then easily be obtained by online UV exposure inside the LAESI ambient ionization source, providing the opportunity to study reactions in real time.

In the present work, we introduce LAESI-MS as an analysis technique in TiO_2_ photocatalysis. The feasibility of the method is demonstrated and critically compared to LC-MS, TiO_2_-μPESI-MS, and DESI-MS TiO_2_ photocatalysis methods for verapamil, buspirone, and testosterone. Furthermore, the method is used to generate reaction products of the selective androgen receptor modulators andarine and ostarine and compared with metabolites obtained in vitro. Finally, time-resolved TiO_2_ photocatalysis LAESI-MS is shown, with the online TiO_2_ photocatalyzed oxidation reaction of verapamil as a model system.

## Experimental

### Materials

TiO_2_-coated glass slides were created by atomic layer deposition of titanium(IV) isopropoxide on glass; the full procedure has been reported elsewhere [20]. Verapamil, buspirone, and titanium(IV) oxide nanopowder P25 were obtained from Sigma-Aldrich (Zwijndrecht, The Netherlands). Testosterone, ostarine, and andarine were kindly donated by RIKILT (Wageningen, The Netherlands). Ultrapure water (H_2_O)—18.2 MΩ × cm^−1^ at 25 °C—was freshly produced daily with a Millipore (Molsheim, France) Integral 3 system. Acetonitrile (ACN) LC-MS grade was bought from VWR (Leuven, Belgium). Methanol (MeOH) UPLC-MS grade was purchased from Biosolve (Valkenswaard, The Netherlands). Formic acid (FA) LC-MS grade was bought from Fisher Scientific (Geel, Belgium) and leucine-enkephalin (leu-enk) for lock mass-corrected mass calibration was purchased from Waters (Manchester, UK).

### LAESI-MS of TiO_2_ Photocatalyzed Oxidation Reactions on Glass Slides

For all compounds, only 1 μL of sample solution (1 mM) was dispensed on TiO_2_-coated glass slides. The sample spots were UV exposed until dry—roughly between 60 and 180 s, depending on solvent volatility—with a Uvet (Guangdong, P.R. China) UV LED Spot Curing System NSC4 (specified maximum peak at 365 nm, intensity 650 mW/cm^2^ at 4 cm distance). Two microliter solvent was added to the dried sample spots before direct analysis with a Protea Biosciences (Morgantown, WV) LAESI DP-1000 system coupled to a Waters Synapt G2S traveling wave ion mobility (IMS) time of flight (TOF) mass spectrometer (MS). LAESI desktop software v.2.0.1.3 (Protea Biosciences) was used to control experimental parameters of the LAESI system. The Nd:YAG optical parametric oscillator mid-infrared laser (2.94 μm) was set to 100% laser power (*Φ* 3.2 J/cm^2^), and 10 pulses with a specified pulse length of 5 ns were acquired on every spot (⌀ 200 μm) at a frequency of 5 Hz. A solution of MeOH/H_2_O (1:1) with 0.1% FA and 40 ng × mL^−1^ leu-enk was used as electrospray solvent in positive ion mode whereas in negative ion mode, a solution of MeOH/H_2_O (1:1) with 100 ng × mL^−1^ leu-enk was used. Electrospray flow rate was 1 μL × min^−1^ and the voltage was set at ~ 3.5 kV (ESI+) or ~ 3.0 kV (ESI−) in order to have a stable ESI signal. The Synapt G2S was controlled by MassLynx v4.1 SCN 883 (Waters) and operated in either positive—for verapamil, buspirone, and testosterone—or negative—for ostarine and andarine—ion TOF-MS resolution mode, *m/z* range 50–1200, scan time 1 s, and source and interface temperatures were both set at 150 °C. Background-subtracted mass spectra were generated using the “combine spectrum” function in MassLynx: five scans, each corresponding to five laser pulses of UV exposed sample spots, were combined and 50 scans, matching laser pulses of non-UV-exposed sample spots (blank) together with electrospray background, were subtracted.

### Time-Resolved TiO_2_ Photocatalysis LAESI-MS

**Figure 1:**
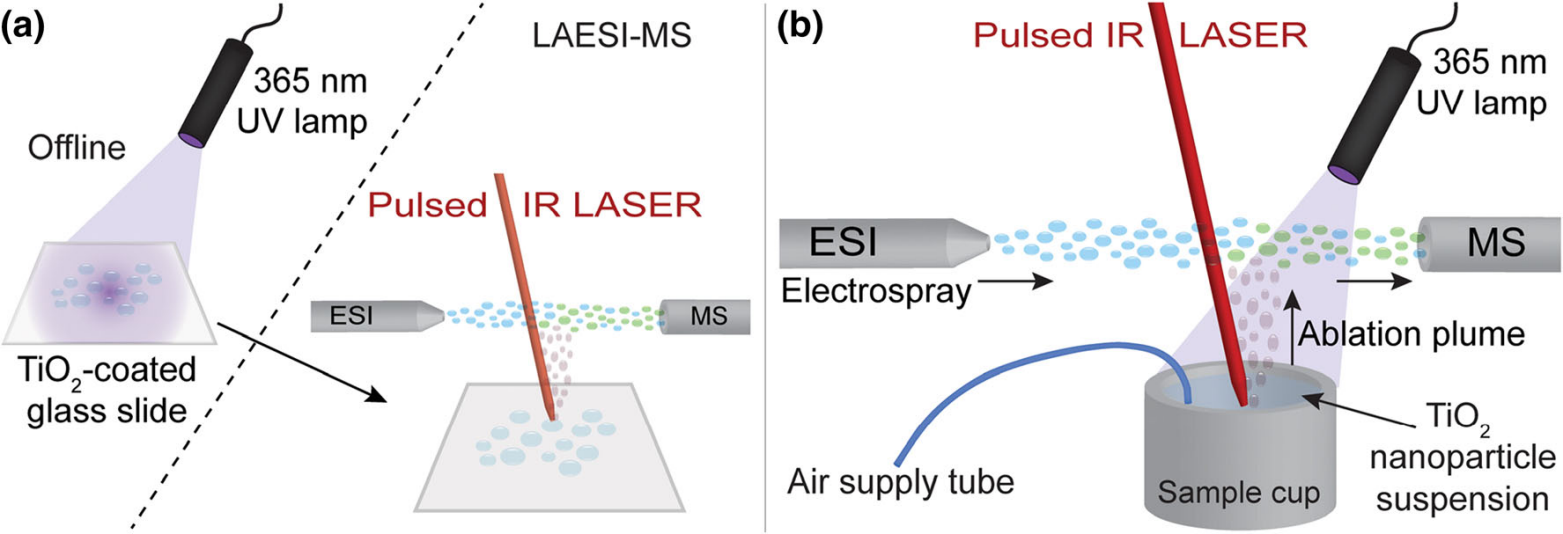
Schematic overview of TiO_2_ photocatalysis LAESI-MS. (**a**) Offline UV exposure of samples on TiO_2_-coated glass slides followed by direct LAESI-MS analysis. (**b**) The online time-resolved TiO_2_ photocatalysis LAESI-MS setup, comprising an ESI probe, a sample cup with PEEK tube for oxygen supply, a mid-IR laser, and a 365 nm UV lamp. Additional details can be found in the experimental section and a picture of (**b**) is shown in Figure S1

For the time-resolved measurements, the UV lamp was installed inside the LAESI system. Verapamil (1 mM) was dissolved in 0.2 g × L^−1^ TiO_2_ nanopowder (H_2_O) and the polypropylene sample cup containing the solution (4 mL) was mounted inside the LAESI system. PEEK tubing, connected to an air supply, was placed into the solution to provide a continuous oxygen source as well as a stirring mechanism due to the bubbling of air (5 mL × min^−1^). An overview of the experimental setup is shown in Figure1b. The sample was online UV exposed for 60 min, during which the mid-IR laser was pulsed continuously at a frequency of 0.1 Hz, and MS scan time was 0.5 s. The heated MS inlet temperature was decreased to 80 °C to minimize solvent evaporation in the sample cup. Extracted ion current (EIC) signals were integrated with MassLynx and the (laser ablation-induced) peak areas obtained were normalized to the verapamil [M+H]^+^ signal.

## Results and Discussion

### LAESI-MS as an Analysis Tool in TiO_2_-Photocatalyzed Oxidation Reactions on Glass Slides

LAESI-MS was examined as a direct analysis tool in TiO_2_ photocatalysis; the generated oxidation products were critically compared with data of previously reported oxidation products of the model compounds verapamil, buspirone, and testosterone [7, 10, 11, 13, 20]. A first notable practical aspect was that LAESI, like DESI, was able to easily analyze samples on surfaces without any sample pre-treatment or lengthy chromatographic separation. Oxidation products were generated by simple offline UV exposure of analyte solutions on TiO_2_-coated glass slides, after which LAESI analysis directly onto the glass slides proved feasible. Additional analyte solution spots on the same glass slides were covered to prevent UV exposure; the obtained LAESI-MS signals of these spots were used for MS background subtraction. Table 1 presents the obtained photocatalyzed oxidation products of verapamil together with their collision-induced dissociation (CID) MS/MS product ions. Detected LAESI-ionized oxidation products at *m/z* 291.206, 441.270, 487.282, and 469.266 correspond to the molecular structures [M-164+H]^+^ (*N*-dealkylation), [M-CH_2_+H]^+^ (demethylation), [M+2O+H]^+^ (dihydroxylation), and [M+O-H_2_+H]^+^ (carbonyl formation), respectively. Both the demethylation (M-CH_2_) and carbonyl formation (M+O-H_2_) appear to occur at various positions in the molecule. For the demethylation, this is shown by the CID product ions *m/z* 151.076 and *m/z* 289.192 (Table 1, patterns B and C, respectively) that both comprise CH_2_ (− 14.02 Da) loss. In the carbonyl formation—oxidation product—this is indicated by the two different CID product ions *m/z* 179.070 and *m/z* 317.187 (Table 1, patterns B and C, respectively), which show a 13.98 Da addition (+O-H_2_) compared to their verapamil equivalents. The observed photocatalyzed oxidation products and CID product ions are similar to those reported in TiO_2_-μPESI and DESI analysis studies [13, 20]. One exception is a product detected with TiO_2_-μPESI-MS at *m/z* 195 (*N*-dealkylation) which was not observed in our data. This absence could be a result of the specific TiO_2_ photocatalysis procedure used, as with TiO_2_-μPESI, the photocatalytic micropillar nanoreactor was integrated in the electrospray ionization chip, which comprises a different setup. Our method has more similarities with the DESI study, in which *m/z* 195 was absent as well [20]. The obtained verapamil CID fragmentation pathway in Table 1 is similar to the one suggested by Walles et al. [30]

**Table 1:** Observed TiO_2_ Photocatalyzed Oxidation Products of Verapamil, as Generated on TiO_2_-Coated Glass Slides, Together with Their CID Product Ions. A Background-Subtracted Mass Spectrum Is Given in Figure S2a and CID MS/MS Spectra of Verapamil and Photocatalyzed Oxidation Products Are Provided in Figure S3

Photocatalytic oxidation product	[M+H]^+^	Observed CID MS/MS product ions (*m/z*)					
		A	B	C	D	E	Other
Verapamil	455.290	150.068	165.091	303.206		260.165	
M-164, N-dealkylation	291.206					260.165	248.152 177.091 165.091 151.076
M-CH_2_	441.270	150.068	165.092 151.076	303.207 289.192	291.207	260.165	
M+2O	487.282		165.092	303.208 289.192		260.165	469.270 440.267
M+O-H_2_	469.266		179.070 165.091 151.076	303.207 317.187		260.166	
				*Proposed CID fragmentation pattern of verapamil*			

For buspirone, the obtained TiO_2_-photocatalyzed oxidation products following LAESI-ionization were *m/z* 402.254, 400.241, 384.240, and 418.253 and are presented along with the their CID MS/MS product ions in Table 2. The obtained oxidation products relate to the molecular structures [M+O+H]^+^ (hydroxylation), [M+O-H_2_+H]^+^ (hydroxylation and dehydrogenation), [M-H_2_+H]^+^ (dehydrogenation), and [M+2O+H]^+^ (dihydroxylation), respectively. As with verapamil, all obtained oxidation products are in excellent agreement with LC-MS and DESI-MS studies on this compound, underlining the feasibility of LAESI-MS as a rapid analysis tool for TiO_2_ photocatalysis [7, 11, 20]. Furthermore, the obtained CID product ions **D** and **F** (Table 2) of photocatalyzed oxidation products M+O (*m/z* 402.254) and M+O-H_2_ (*m/z* 400.241) in particular demonstrate distinctive CID fragmentation. The hydroxylation (M+O) product suggests a rapid loss of H_2_O upon CID, making the hydroxylation site undetected in CID MS/MS analysis from the *m/z* values of fragment ions **D** and **F**. However, with the hydroxylation and dehydrogenation (M+O-H_2_) product, this site is indicated to be in CID product ion **F** due to the increased *m/z* value of this CID product ion (from *m/z* 265 to 279). The dehydrogenation site is located in the piperazine moiety, as indicated by CID MS/MS product ion **E** (*m/z* 162.090, Table 2). The opportunity to include CID MS/MS is a useful option, but a minor shortcoming of ambient ionization MS compared to hyphenated-MS techniques is the absence of the extra separation dimension, e.g., there were four chromatographic peaks reported by Calza et al. in the LC-MS analysis of hydroxy-buspirone (*m/z* 402), corresponding to at least four different isobaric hydroxylation products [7]. An additional separation dimension compatible with ambient ionization MS is ion mobility (IMS) [26]. The applicability of IMS was investigated by calculation of collision cross sections (CCS) for reported isobaric hydroxylation products and is provided in Table S1 [7]. Buspirone hydroxylation products are varying in CCS by circa 1%, whereas the IMS resolving power of used instrumentation is capable of separating compounds with CCS differences of over approximately 5% [31]. The IMS separation of buspirone hydroxylation products is therefore beyond the resolving power of present instrumentation and was not further investigated here. Nonetheless, studies comprising other molecules and/or different ion mobility hardware could benefit from separation with this technique. The CID MS/MS fragmentation pathway as proposed in Table 2 is in good agreement with literature [7, 32].

**Table 2:** Observed TiO_2_-Photocatalyzed Oxidation Products of Buspirone, and Their CID MS/MS Product Ions. A Background-Subtracted Mass Spectrum Is Given in Figure S2b and CID MS/MS Spectra of Buspirone and Photocatalyzed Oxidation Products Are Provided in Figure S4

Photocatalytic oxidation product	[M+H]^+^	Observed CID MS/MS product ions (*m/z*)							
		A	B	C	D	E	F	G	Other
Buspirone	386.255	222.149		150.103	122.072		265.191		
M+O	402.254	222.149	251.176		122.072		265.192		384.240
M+O-H_2_	400.241				122.072		279.170	150.067	372.240
M-H_2_	384.240	222.149				162.090			
M+2O	418.253				122.072			150.067	177.114 330.192 372.239 384.239
					*Proposed CID fragmentation pattern of buspirone*				

Finally, the steroid testosterone was studied to show the feasibility of TiO_2_ photocatalysis LAESI-MS for analysis of a more hydrophobic compound. Testosterone was dissolved in H_2_O/ACN (1:1 *v*/*v*) prior to the analysis. ACN is known to be an OH radical scavenger and may therefore somewhat hamper the TiO_2_-photocatalyzed oxidation of testosterone [10, 33]. Despite this organic modifier, oxidation products were easily obtained and are presented with their CID MS/MS product ions in Table S2. The observed reaction products M+O (*m/z* 305.209), M+O-H_2_ (*m/z* 303.194), and M-H_2_ (*m/z* 287.203) are in line with the products detected in conventional LC-MS analysis [10, 11].

### TiO_2_ Photocatalysis LAESI-MS of Selective Androgen Receptor Modulators on Glass Slides

**Figure 2:**
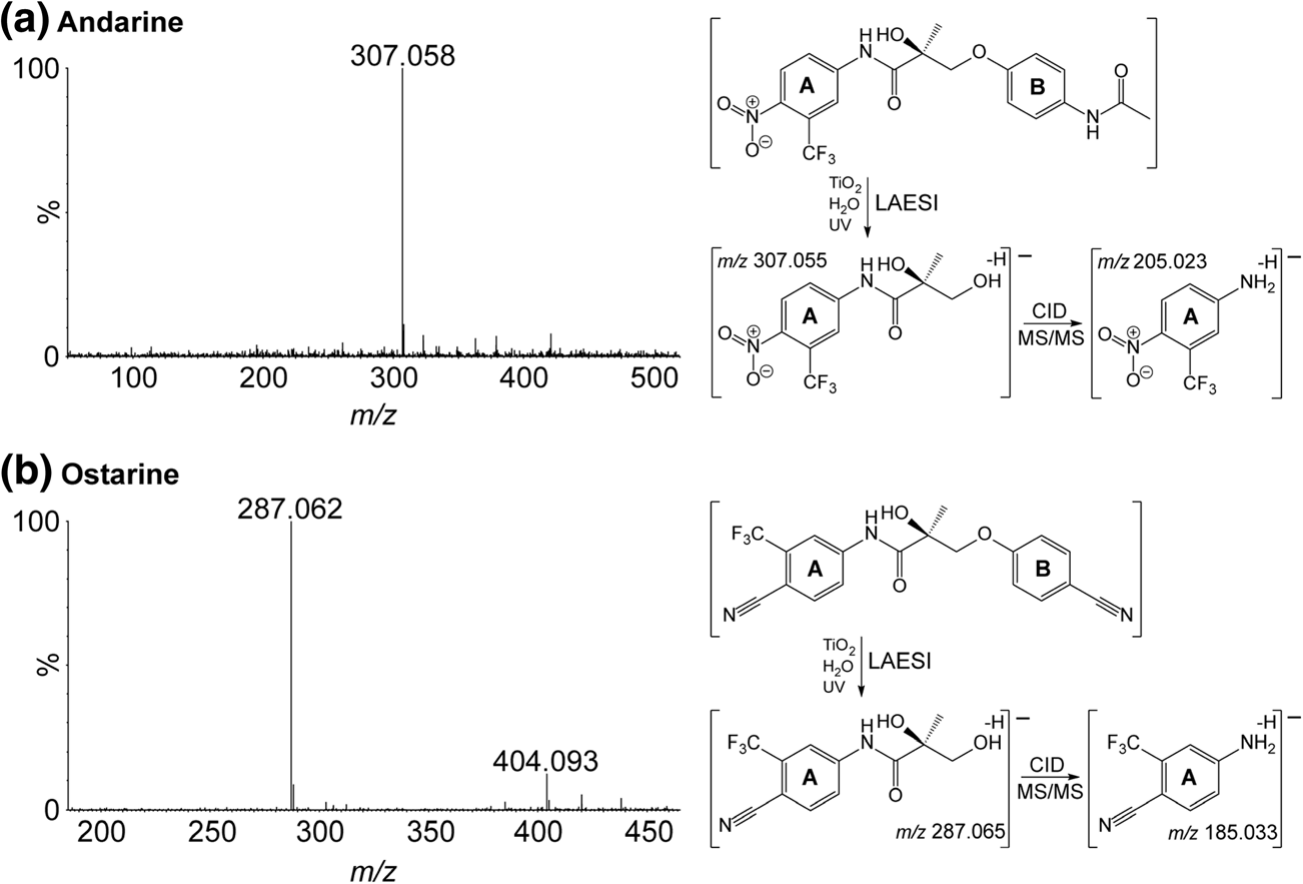
Background-subtracted mass spectra of the oxidation products of andarine (**a**) and ostarine (**b**), with their proposed structures. Obtained CID MS/MS spectra are provided in Figures S7 (ostarine product, *m/z* 404.093), S8 (andarine product, *m/z* 307.058), and S9 (ostarine product, *m/z* 287.062)

Following the verification of LAESI-MS as a suitable analysis tool in TiO_2_ photocatalysis on glass slides, the method was used to study the oxidation products of the selective androgen receptor modulators andarine and ostarine. The only photocatalytic reaction product of andarine was observed at *m/z* 307.058 (Figure 2a) following LAESI-MS analysis, and the proposed mechanism is depicted in Figure S6. The proposed structure could further be confirmed by MS/MS of *m/z* 307.058, via the observation of a fragment ion at *m/z* 205.023. This *m/z* 307.058 peak and similar MS/MS spectra were reported to belong to the most abundant metabolite in several biological in vitro and in vivo studies, using human liver microsomes and human urine [34–40]. In those studies, reported phase II metabolites produced by enzymes, such as glutathione-S-transferase, N-acetyl transferase, and sulfotransferase, are obviously not observed in TiO_2_-photocatalyzed oxidation studies.

In case of ostarine, the LAESI-ionized oxidation products were observed at *m/z* 287.062 and *m/z* 404.093 (Figure 2b). The ion at *m/z* 287.062 is proposed to be produced following a similar reaction mechanism (Figure S6) as *m/z* 307.058 of andarine. The structure is further confirmed by MS/MS to yield a CID fragment ion at *m/z* 185.033, which is in agreement with literature [40, 41]. The oxidation product appearing at *m/z* 404.093 is most likely formed by hydroxylation of ostarine. Multiple hydroxylation sites were identified by MS/MS experiments. The most obvious sites are aromatic rings (Figure S7). The CID fragment ions at *m/z* 285.049 and *m/z* 118.030 correspond to hydroxylation in aromatic ring **A**, whereas the fragment ions at *m/z* 269.054 and *m/z* 134.025 associate with hydroxylation at ring **B**. A fragmentation mechanism for these compounds has been reported previously [33]. However, we here propose a different CID fragmentation mechanism, leading to fragment ions with a stable C-N bond instead of a labile N-O bond (Figure S7b). These results demonstrate that our method provides rapid identification of photocatalyzed oxidation products that mimic products found in several in vitro studies [34–41].

Although both andarine and ostarine do not significantly absorb UV light at *λ* = 365 nm, the same experiment, but on regular—uncoated—glass slides was performed to exclude any possibility of photodegradation due to direct UV exposure. Indeed, no reaction products were obtained (Figure S10). Please note that all compounds used in this study do not significantly absorb UV light at *λ* > 300 nm and the used UV lamp does not emit UV light at *λ* < 350 nm (Figure S11). It is therefore unlikely that any products shown in this work are a result of photodegradation.

### Time-Resolved Photocatalyzed Oxidation LAESI-MS of Verapamil Using TiO_2_ Nanoparticle Suspensions

**Figure 3:**
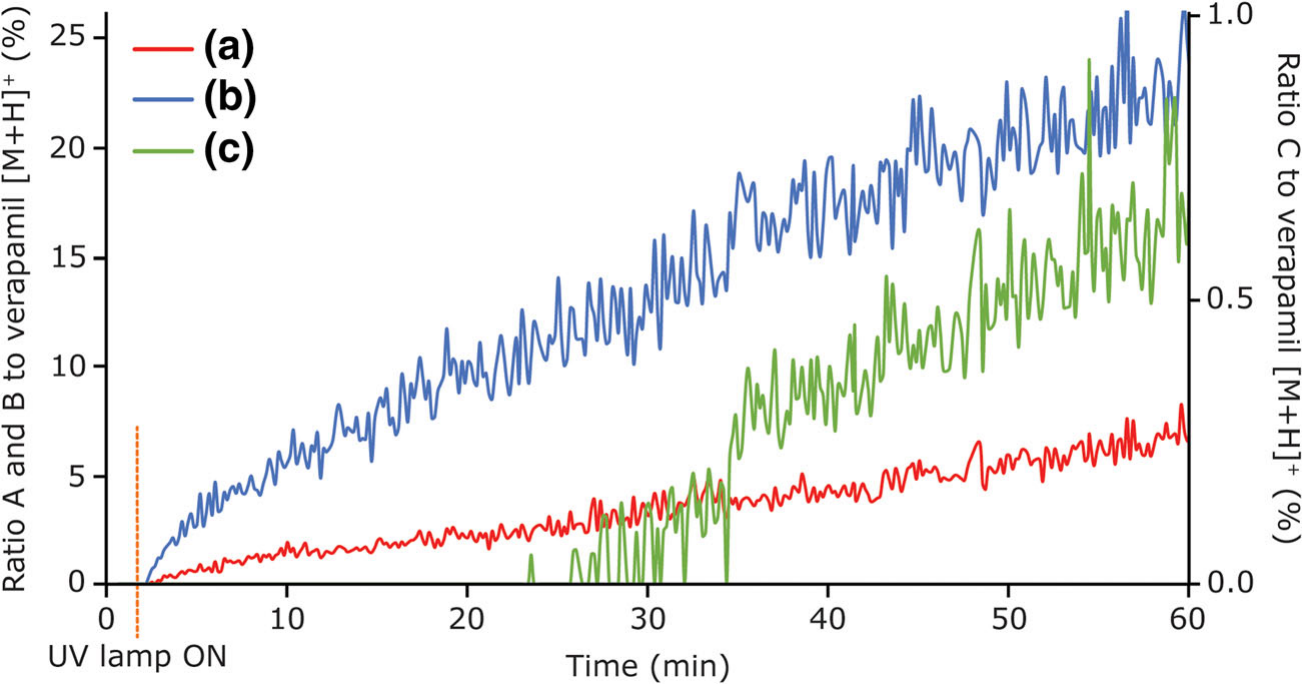
Time-resolved photocatalyzed oxidation LAESI-MS of verapamil using a TiO_2_ nanoparticle suspension. (**a**) The formation of the *N*-dealkylation product (*m/z* 291.21) of verapamil. (**b**) The formation of the demethylation product of verapamil (*m/z* 441.28). (**c**) The product of both the *N*-dealkylation and demethylation (*m/z* 277.19), which starts to appear around ~ 25 min (intensity shown at secondary *y*-axis). The 365-nm UV lamp was switched ON after 2 min. All lines were normalized to verapamil [M+H]^+^. Data were averaged (*n* = 3, for clarity, error bars are not shown), individual data are shown in Figure S14

In order to monitor the generation of reaction products in time, an online setup was created by installing a reaction cup and the UV lamp inside the LAESI system (Figures 1b and S1). Besides measuring oxidation products from TiO_2_-coated glass slides as demonstrated in the previous paragraphs, LAESI is also capable of measuring those directly from a sample cup containing suspended TiO_2_ nanoparticles in water. Verapamil was used as a model compound and the generation of several products during a reaction time of 1 h is shown in Figure 3. After switching on the 365-nm UV lamp, at 2-min runtime, almost instantly two major products of verapamil, the *N*-dealkylation and demethylation products (Figure 3, signals a and b) were observed. These reaction products steadily increased in abundance during the entire analysis runtime of 60 min (Figure 3, raw data in Figure S12). The added value of time-resolved analysis started to show after ca. 25-min reaction time, when a product ion at *m/z* 277.19 started to appear (Figure 3, signal c). This *m/z* value is the result of both *N*-dealkylation and demethylation of verapamil and clearly demonstrates the power of this system in identifying subsequent oxidation steps. In addition, also the product of a double demethylation (*m/z* 427.26) was observed after ca. 18 min (Figure S13). Following these results, this time-resolved method allows the optimization of reaction conditions preliminary to high-throughput static experiments. Despite the inherent poor reproducibility of MS data from individual LAESI pulses, the reproducibility of time-resolved TiO_2_ photocatalyzed LAESI-MS over three different analyses is actually quite good, cf. the individual lines of the (*n* = 3) averaged data presented in Figure 3 given in Figure S14. This indicates that time-resolved analysis of the oxidation products of drugs is not only feasible by LAESI, but also easily adds mechanistic information that is not available upon analysis of the composition of just one time point.

## Conclusions

We have developed an ambient TiO_2_ photocatalysis LAESI MS method to generate photocatalyzed oxidation products of drugs that are similar to phase I metabolites. Like other ambient ionization MS approaches the concept features rapid simplified analysis without any sample pretreatment or lengthy chromatographic separations. In addition, LAESI MS was able to measure drugs and their metabolites directly from the surface of TiO_2_-coated glass slides as well as from TiO_2_ nanoparticle suspensions. Among others, the method on TiO_2_-coated glass slides was demonstrated with the selective androgen receptor modulators andarine and ostarine to yield oxidation reaction products similar to those obtained in various in vitro studies. Moreover, a novel fully integrated, time-resolved LAESI MS photocatalyzed oxidation approach was successfully developed using a TiO_2_ nanoparticle suspension and demonstrated for verapamil. The time-resolved TiO_2_ photocatalysis LAESI MS exhibited excellent stability and enabled the monitoring of reaction products during a reaction time of at least 1 h. This method can be used in future research to expeditiously assess drug candidates in the early stages of development as well as an online tool for time-resolved monitoring for this and of other—non-TiO_2_ based—reactions.

## Electronic supplementary material

ESM 1 (DOCX 10565 kb)
