# Supplementary material for: TiO2 Photocatalyzed Oxidation of Drugs Studied by Laser Ablation Electrospray Ionization Mass Spectrometry
Source: J Am Soc Mass Spectrom. 2019 Jan 7;30(4):639–46. doi: 10.1007/s13361-018-2120-x (PMC6445813; doi:10.1007/s13361-018-2120-x)
Supplement: Supplementary file 1 — (DOCX 10565 kb) [file 13361_2018_2120_MOESM1_ESM.docx]

# TiO_2_ Photocatalyzed Oxidation of Drugs Studied by Laser Ablation Electrospray Ionization Mass Spectrometry

Fred A. M. G. van Geenen,^1,2^ Maurice C. R. Franssen,^1^ Ville Miikkulainen,^3^ Mikko Ritala,^3^ Han Zuilhof,^1,4,5^ Risto Kostiainen,^6^ Michel W. F. Nielen^1,7^

^1^ Laboratory of Organic Chemistry, Wageningen University, Stippeneng 4, 6708 WE Wageningen, The Netherlands

^2^ TI-COAST, Science Park 904, 1098 XH Amsterdam, The Netherlands

^3^ Department of Chemistry, University of Helsinki, P.O. Box 55, FI-00014 Helsinki, Finland

^4^ School of Pharmaceutical Sciences and Technology, Tianjin University, 92 Weijin Road, Tianjin, P.R. China

^5^ Department of Chemical and Materials Engineering, King Abdulaziz University, Jeddah, Saudi Arabia

^6^ Drug Research Program, Division of Pharmaceutical Chemistry and Technology, Faculty of Pharmacy, University of Helsinki, P.O. Box 56, FI-00014 Helsinki, Finland

^7^ RIKILT, Wageningen University & Research, P.O. Box 230, 6700 AE Wageningen, The Netherlands

*Correspondence to:* Michel Nielen; *E-mail:* Michel.nielen@wur.nl

**Table of Contents**

Page

Figure S1. Picture of the time-resolved LAESI-MS setup depicted in Figure 1B 3

Figure S2. TiO_2_ photocatalyzed oxidation LAESI-MS background subtracted mass spectra of A) verapamil, B) buspirone and C) testosterone on TiO_2_-coated glass slides 4

Figure S3. CID MS/MS mass spectra of verapamil (panel A) and observed TiO_2_ photocatalyzed oxidation products (panels B-E) 5

Table S1. Structures and calculated collision cross sections of buspirone hydroxylation products 6

Figure S4. CID MS/MS mass spectra of buspirone (panel A) and observed TiO_2_ photocatalyzed oxidation products (panels B-E) 7

Table S2. Observed photocatalyzed oxidation products of testosterone, as generated on TiO_2_-coated glass slides together with their CID MS/MS product ions 8

Figure S5. CID MS/MS mass spectra of testosterone (panel A) and observed TiO_2_ photocatalyzed oxidation products (panels B-D) 9

Figure S6. Proposed mechanism for TiO_2_ photocatalyzed oxidation products of andarine and ostarine, observed at *m/z* 307.058 and *m/z* 287.062, respectively 9

Figure S7. MS/MS on ostarine hydroxylation product ions with proposed structures 10

Figure S8. Andarine MS/MS on *m/z* 307 11

Figure S9. Ostarine MS/MS on *m/z* 287 11

Figure S10. Background subtracted mass spectra of Ostarine and Andarine on glass slides without TiO_2_ coating 11

Figure S11. Emission spectrum of used UV lamp 12

Figure S12. EIC’s of verapamil and oxidation products generated by time-resolved photocatalysis LAESI-MS using suspended TiO_2_ nanoparticles 13

Figure S13. EIC of *m/z* 427.26 following time-resolved TiO_2_ photocatalyzed oxidation LAESI-MS of verapamil 14

Figure S14. Individual data for oxidation products generated by time-resolved TiO_2_ photocatalysis LAESI-MS as presented in Figure 3 15

References 16


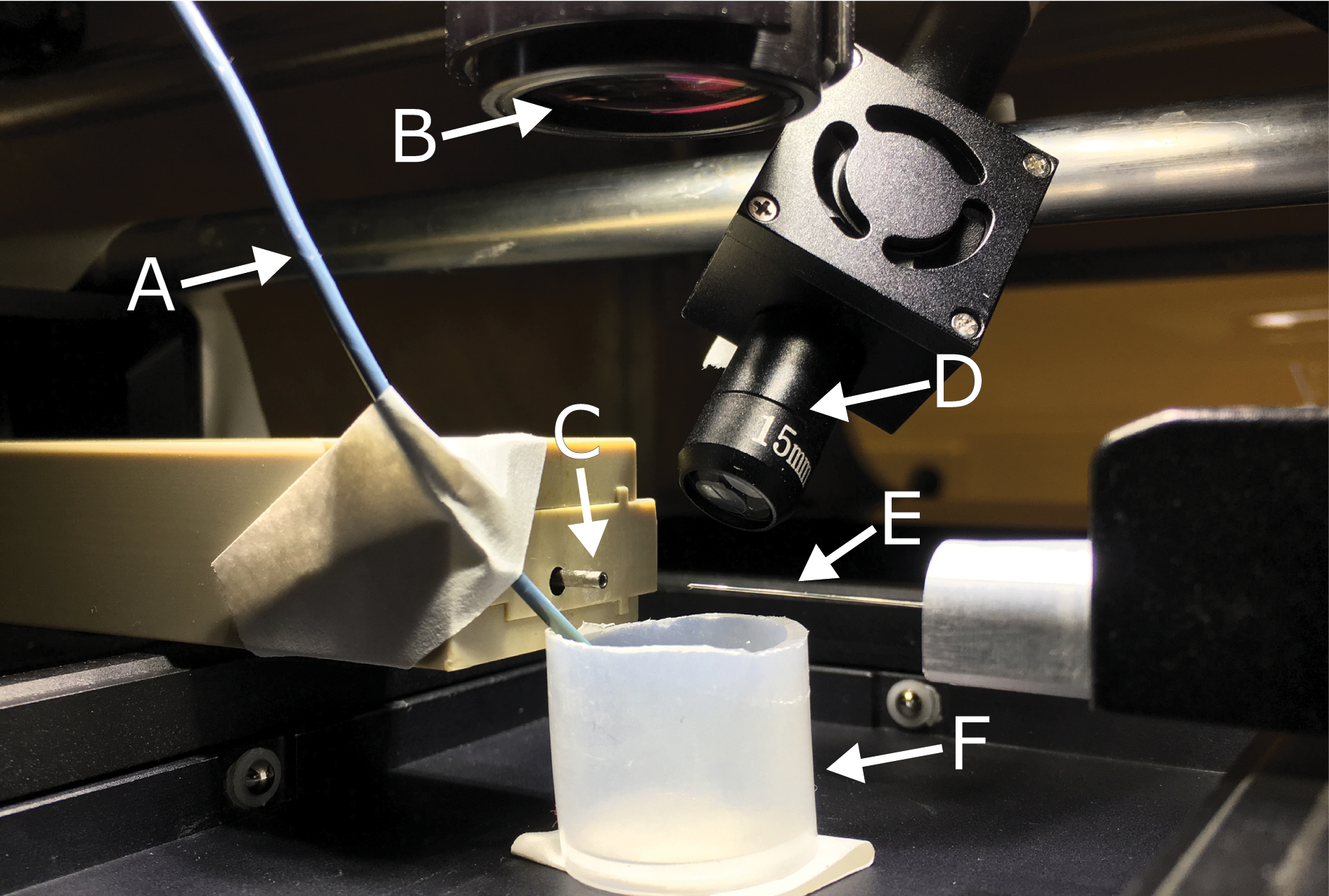


Figure S1. Picture of the time-resolved LAESI-MS setup depicted in Figure 1B

. **A** PEEK tubing for bubbling air (oxygen supply and stirring mechanism), **B** mid-IR laser focussing lens, **C** heated MS inlet, **D** 365 nm UV lamp, **E** ESI spray needle and **F** polypropylene sample cup. The distance between MS inlet **C** and the ESI needle tip **E** was 15 mm.


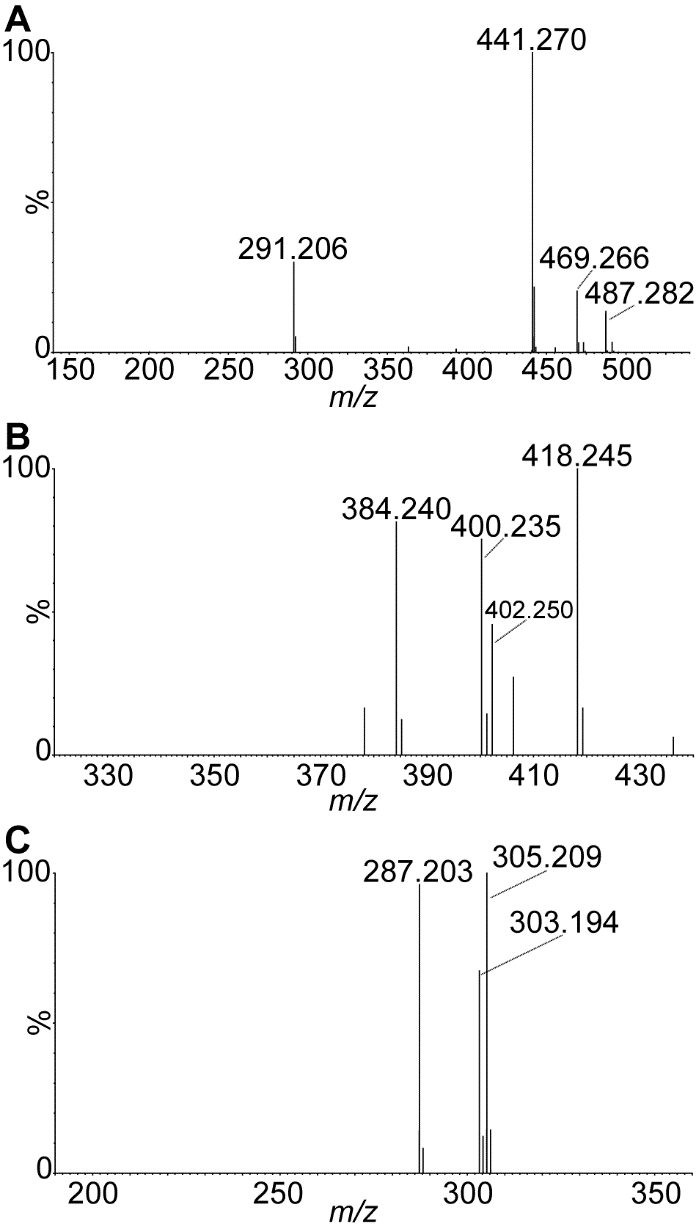


Figure S2. TiO_2_ photocatalyzed oxidation LAESI-MS background subtracted mass spectra of A) verapamil, B) buspirone and C) testosterone on TiO_2_-coated glass slides

. CID MS/MS mass spectra of observed product ions are presented in Figures S3 (verapamil), S4 (buspirone) and S5 (testosterone). For conditions, see experimental section.


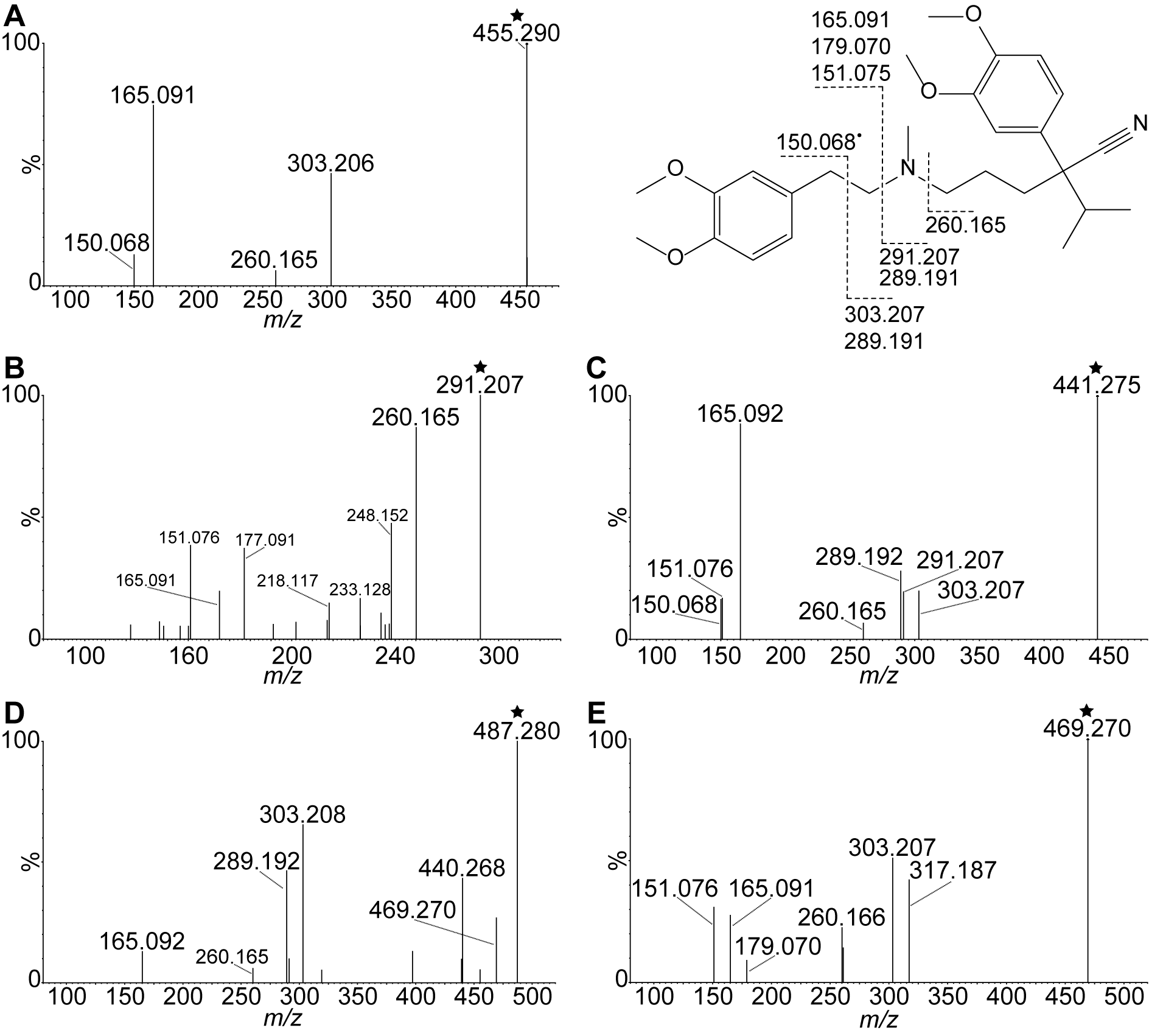


Figure S3. CID MS/MS mass spectra of verapamil (panel A) and observed TiO_2_ photocatalyzed oxidation products (panels B-E)

. Selected precursor ion is annotated with a star. The fragmentation pattern is in excellent agreement with literature. [1, 2]

Table S1. Structures and calculated collision cross sections of buspirone hydroxylation products

. Structures of hydroxylation products were proposed by Calza et al. [3] Hydroxy buspirone 3D structures were auto optimized using Avogadro V1.1.1 (<http://avogadro.cc/>) with force field set to MMFF94s. CCS values were calculated using CCSCalc (Waters), gas radius was 1.0 Å and CCS tolerance 0.1%.

| Structure | CCS (Ω) | Deviation (%) from average |
| --- | --- | --- |
|  | 149.9 | 0.8% |
|  | 149.6 | 0.6% |
|  | 148.4 | 0.2% |
|  | 146.8 | 1.3% |


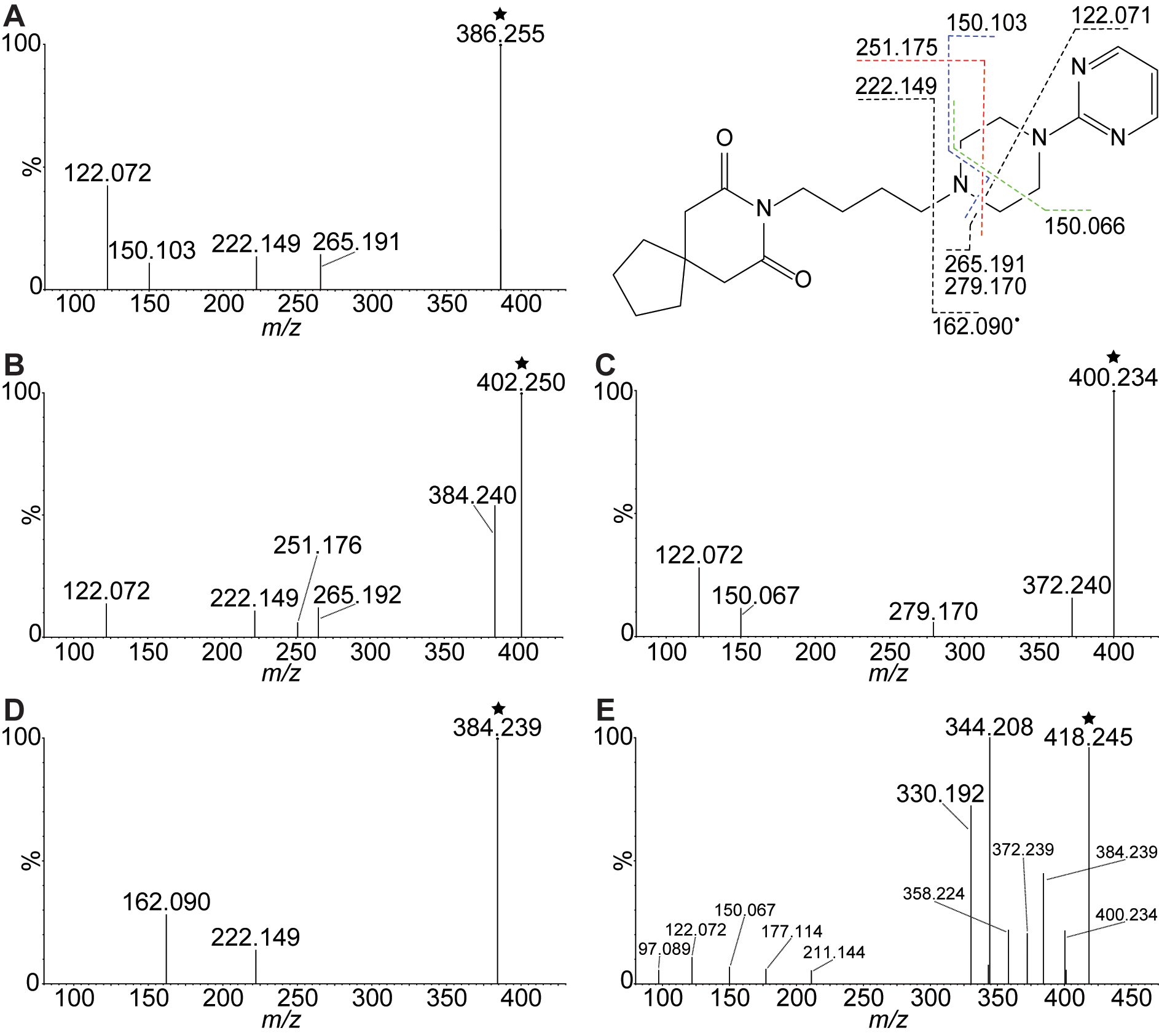


Figure S4. CID MS/MS mass spectra of buspirone (panel A) and observed TiO_2_ photocatalyzed oxidation products (panels B-E)

. Selected precursor ion is annotated with a star. The fragmentation pattern is in excellent agreement with literature. [3, 4]

Table S2. Observed photocatalyzed oxidation products of testosterone, as generated on TiO_2_-coated glass slides together with their CID MS/MS product ions

. A background subtracted mass spectrum is given in Figure S2C and MS/MS spectra of testosterone and observed oxidation products are provided in Figure S5.

| Photocatalyzed oxidation product | [M+H]^+^ | Observed CID MS/MS product ions (m/z) | | | | |
| --- | --- | --- | --- | --- | --- | --- |
|  |  | A | | B | other | |
| Testosterone | 289.216 | 109.065 | | 97.065 | 253.195 | 271.205 |
| M-H_2_ | 287.203 | 109.062 | | 97.064 | 251.179 | 269.193 |
| M+O-H_2_ | 303.194 | 109.062 | | 97.064 |  | 267.173 |
| M+O | 305.209 | 109.064 | | 97.064 |  | 269.185 |
|  | | | 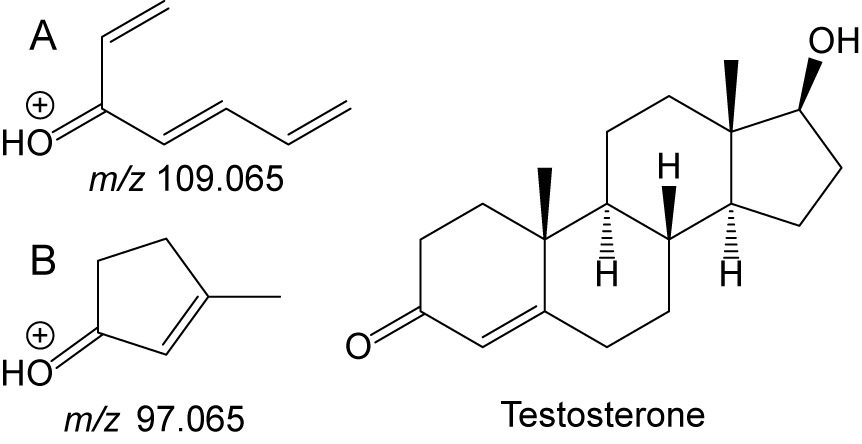  *Proposed m/z 97 and 109 CID product ions of testosterone* | | | |


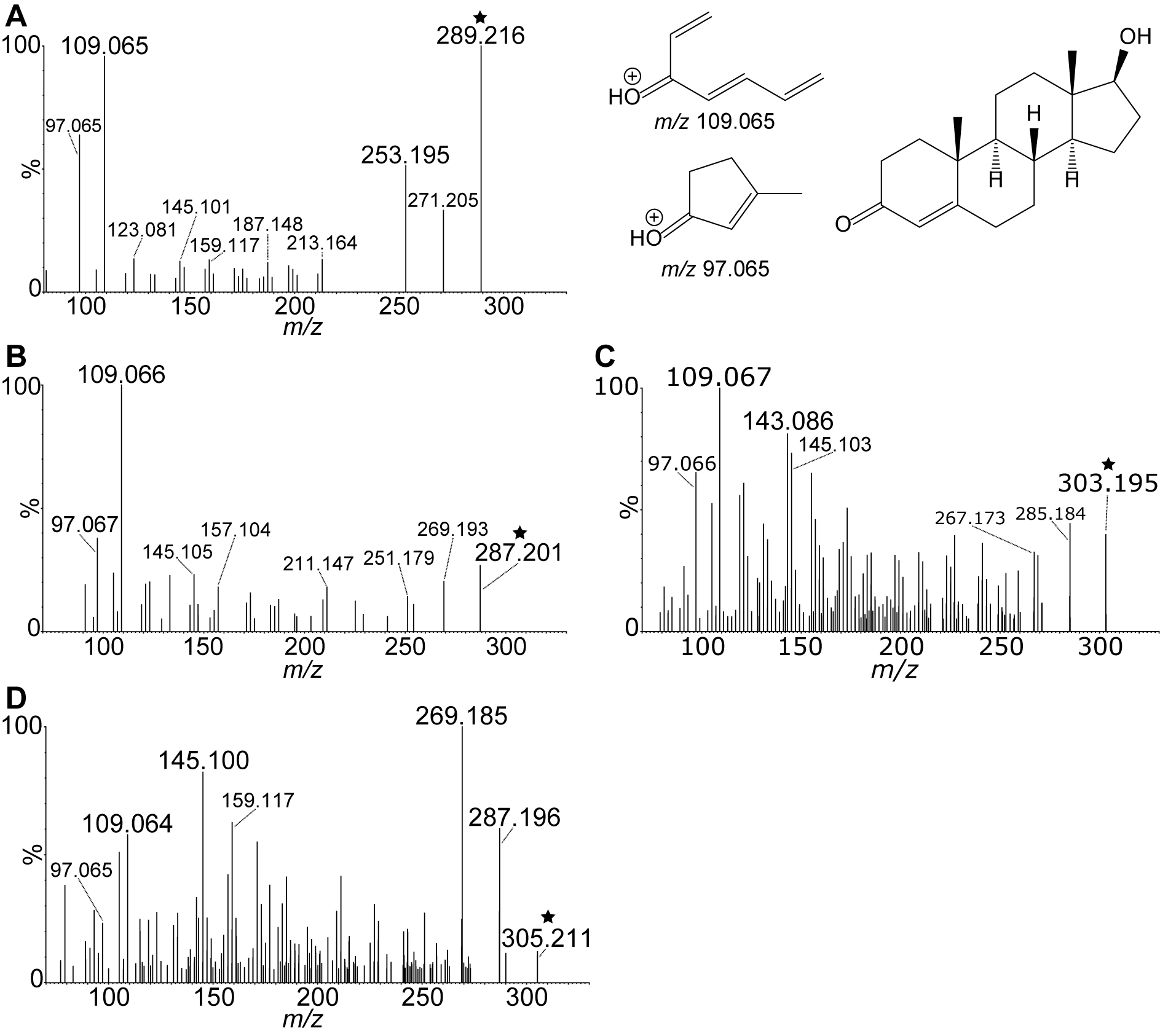


Figure S5. CID MS/MS mass spectra of testosterone (panel A) and observed TiO_2_ photocatalyzed oxidation products (panels B-D)

. Selected precursor ion is annotated with a star. The structures for *m/z* 97 and *m/z* 109 are in agreement with literature. [5, 6]


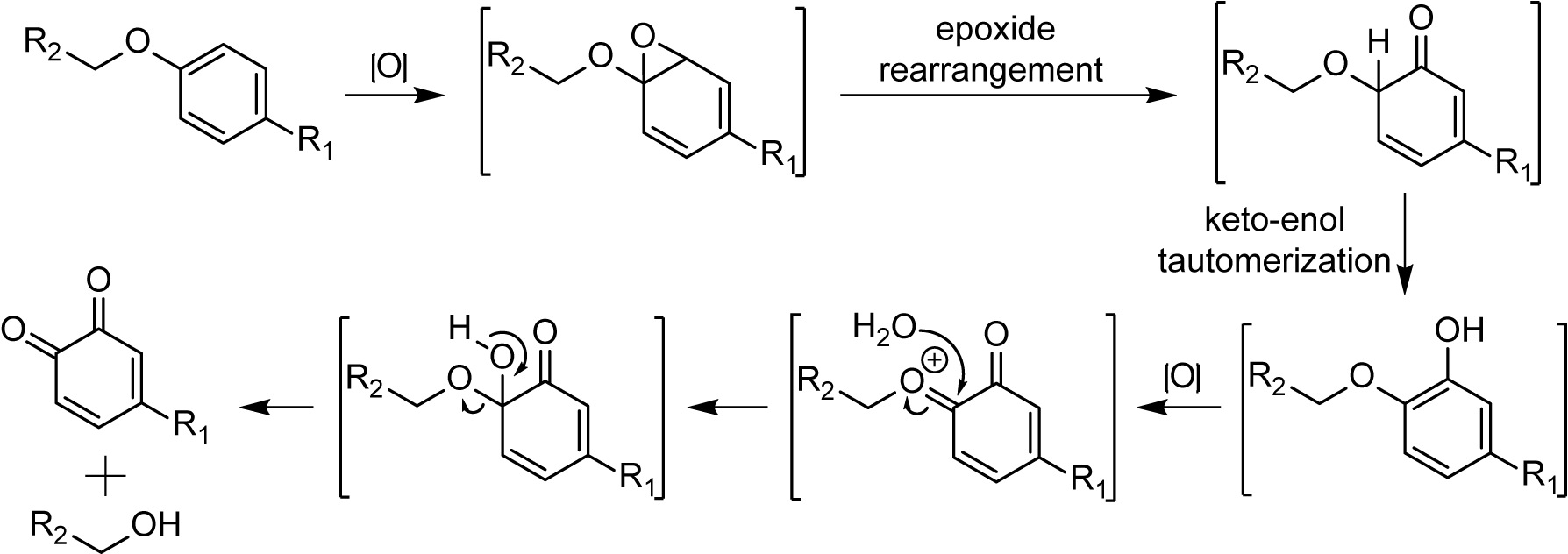


Figure S6. Proposed mechanism for TiO_2_ photocatalyzed oxidation products of andarine and ostarine, observed at *m/z* 307.058 and *m/z* 287.062, respectively

.


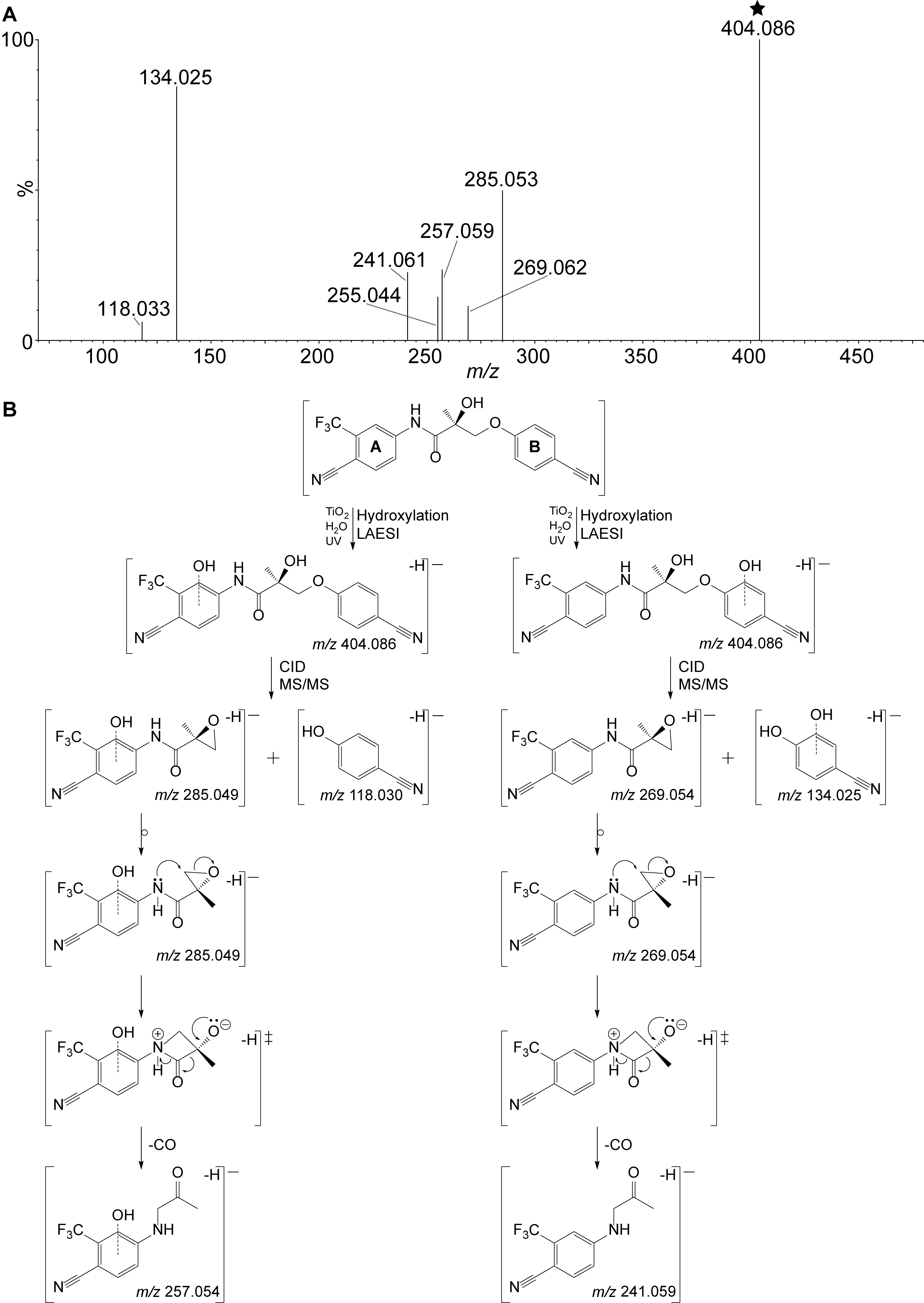


Figure S7. MS/MS on ostarine hydroxylation product ions with proposed structures

. We proposed an alternative fragmentation mechanism to form *m/z* 257 (similarly 241) than which has been proposed previously. [7]


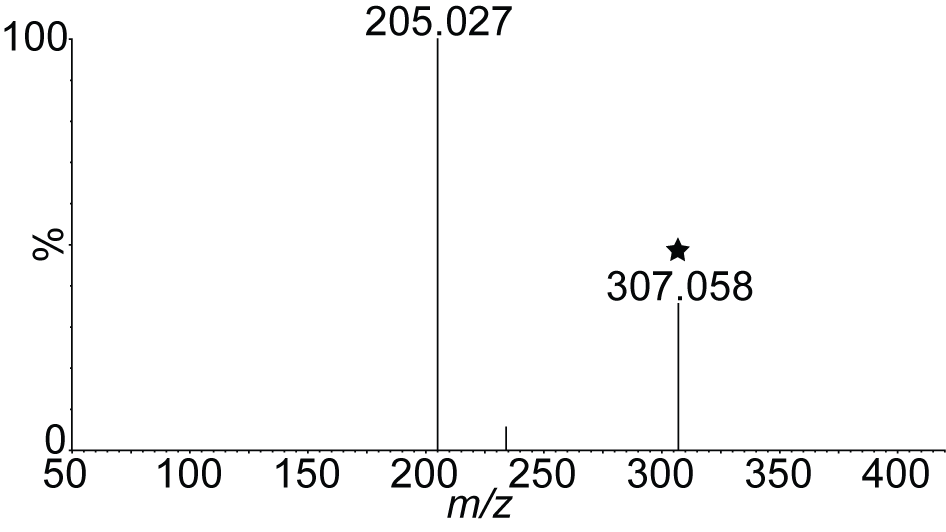


Figure S8. Andarine MS/MS on *m/z* 307

. Selected precursor ion is annotated with a star.


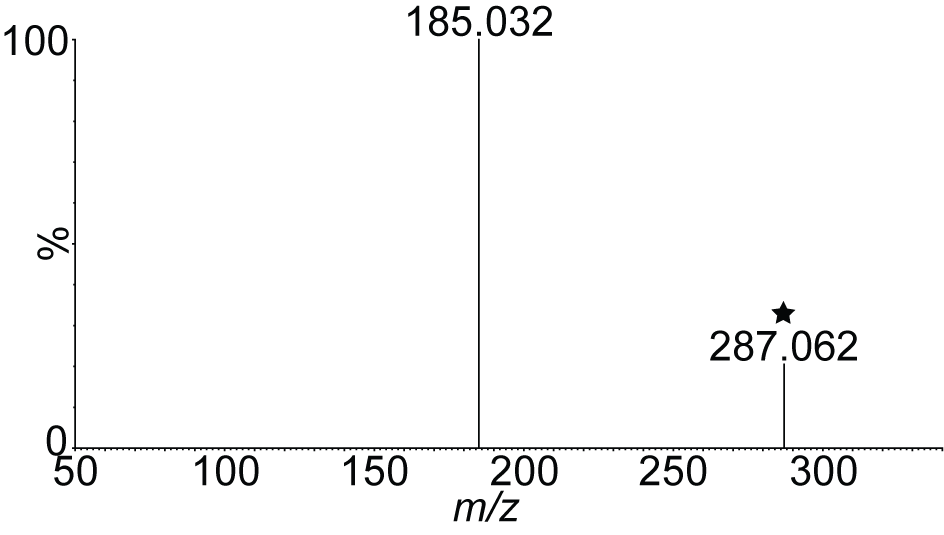


Figure S9. Ostarine MS/MS on *m/z* 287

. Selected precursor ion is annotated with a star.


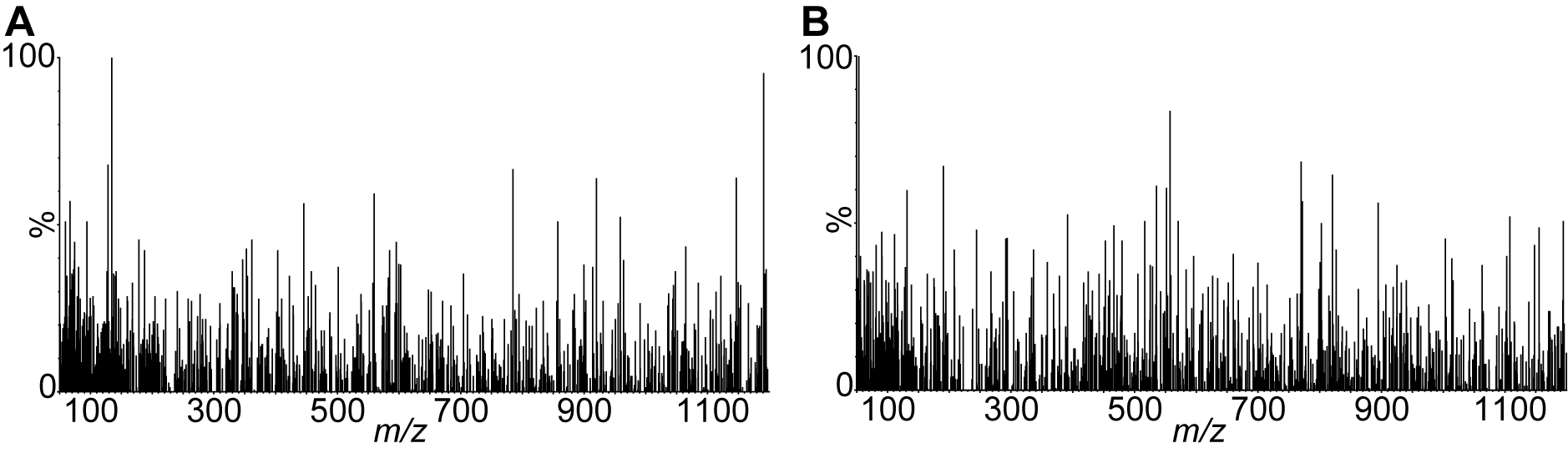


Figure S10. Background subtracted mass spectra of Ostarine and Andarine on glass slides without TiO_2_ coating

. Both samples – Figures A (Ostarine) and B (Andarine) – were treated as described in the experimental section. This experiment was meant to detect any effect of photodegradation as a result of uncatalyzed UV exposure; yet, no effect (product) was observed.


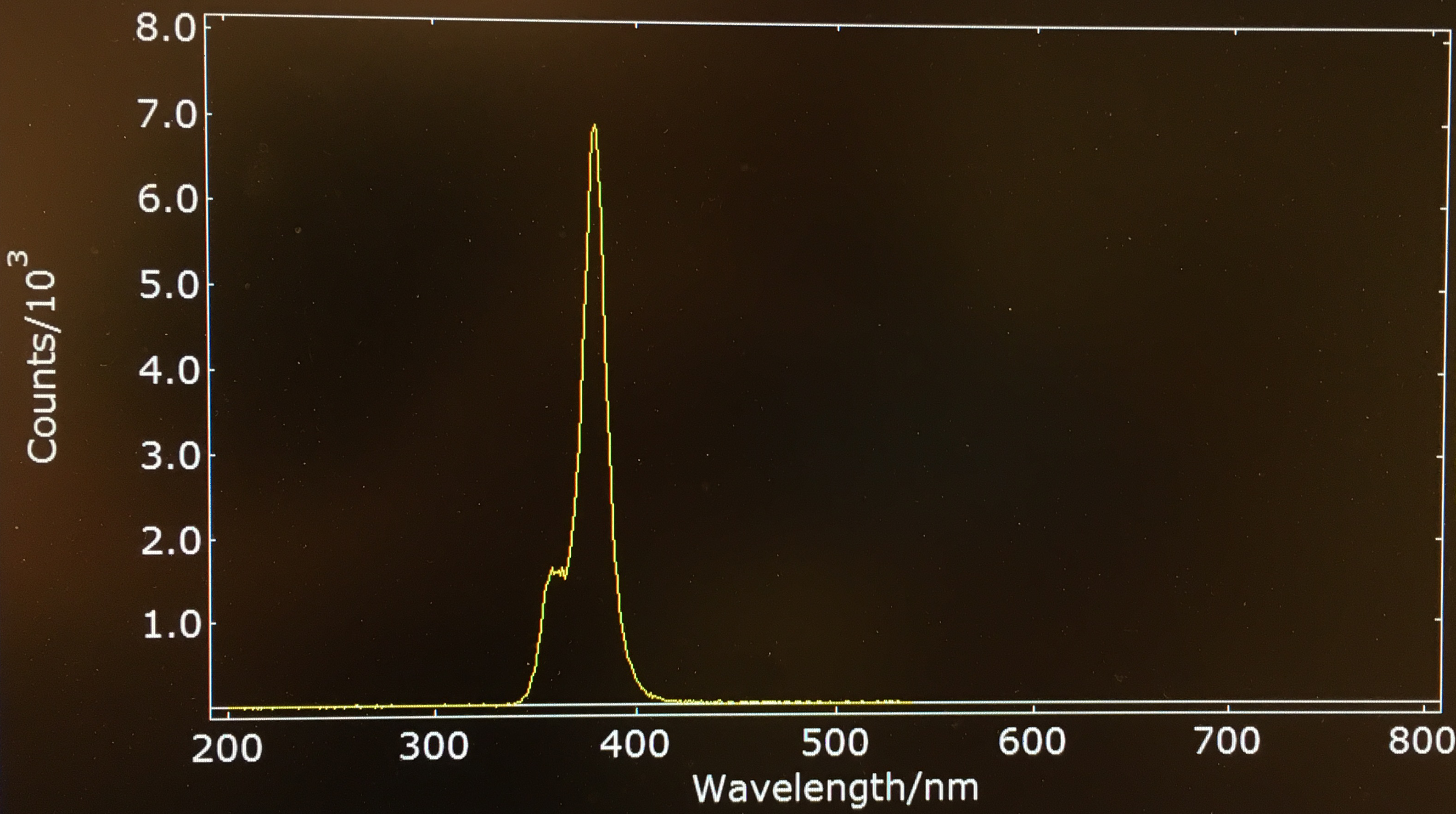


Figure S11. Emission spectrum of used UV lamp

.


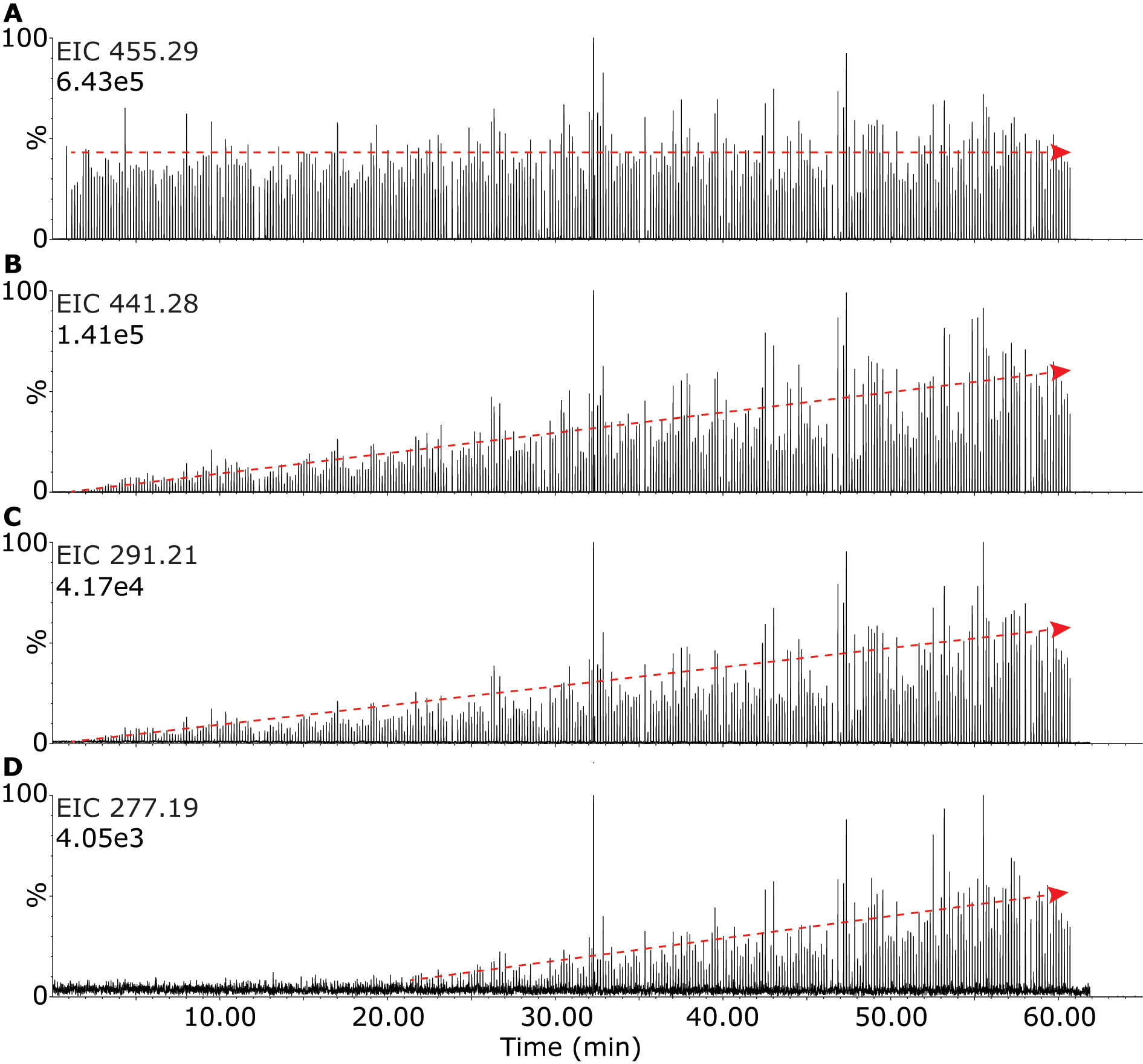


Figure S12. EIC’s of verapamil and oxidation products generated by time-resolved photocatalysis LAESI-MS using suspended TiO_2_ nanoparticles

. Each peak in the EIC’s is a result of a laser pulse (injection) with a frequency of 0.1 Hz. The red lines indicate the trend in the raw data. **A** shows EIC of verapamil [M+H]^+^ which shows a constant signal (apart from normal instrumental – LAESI – variation) for 60 minutes reaction time. **B** and **C** show the TiO_2_ photocatalysis products [M-CH_2_+H]^+^ (demethylation) and [M-164+H]^+^ (N-dealkylation), respectively. Finally, **D** shows the product of both the N-dealkylation and demethylation, which are depicted in **B** and **C,** and is observed starting from ~25 minutes onwards. This result shows consecutive reactions of already formed products and demonstrates the relevance of time-resolved measurements.


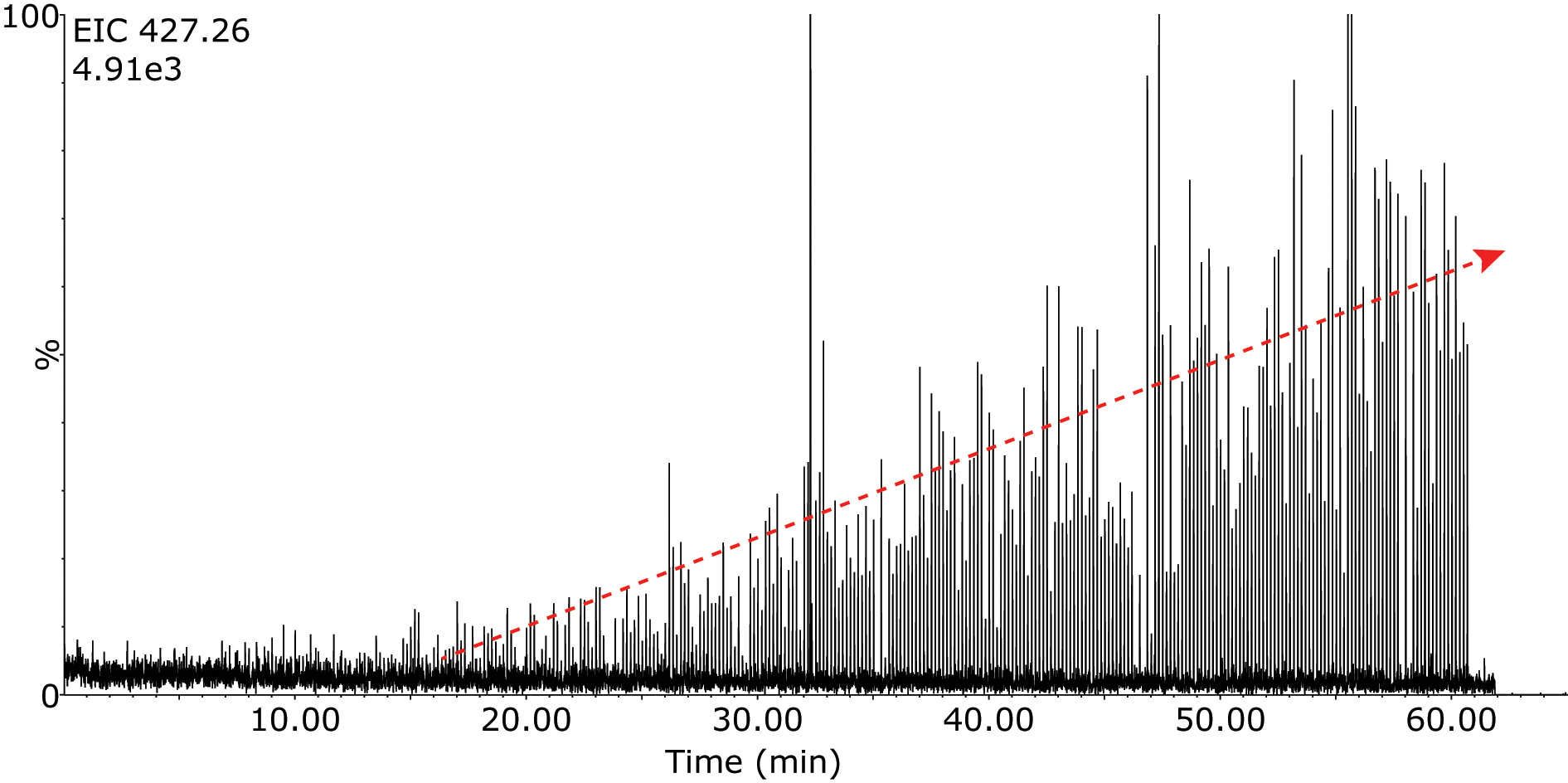


Figure S13. EIC of *m/z* 427.26 following time-resolved TiO_2_ photocatalyzed oxidation LAESI-MS of verapamil

. Each peak in the EIC is a result of a laser pulse (injection) with a frequency of 0.1 Hz. The red line indicates the trend in the raw data.


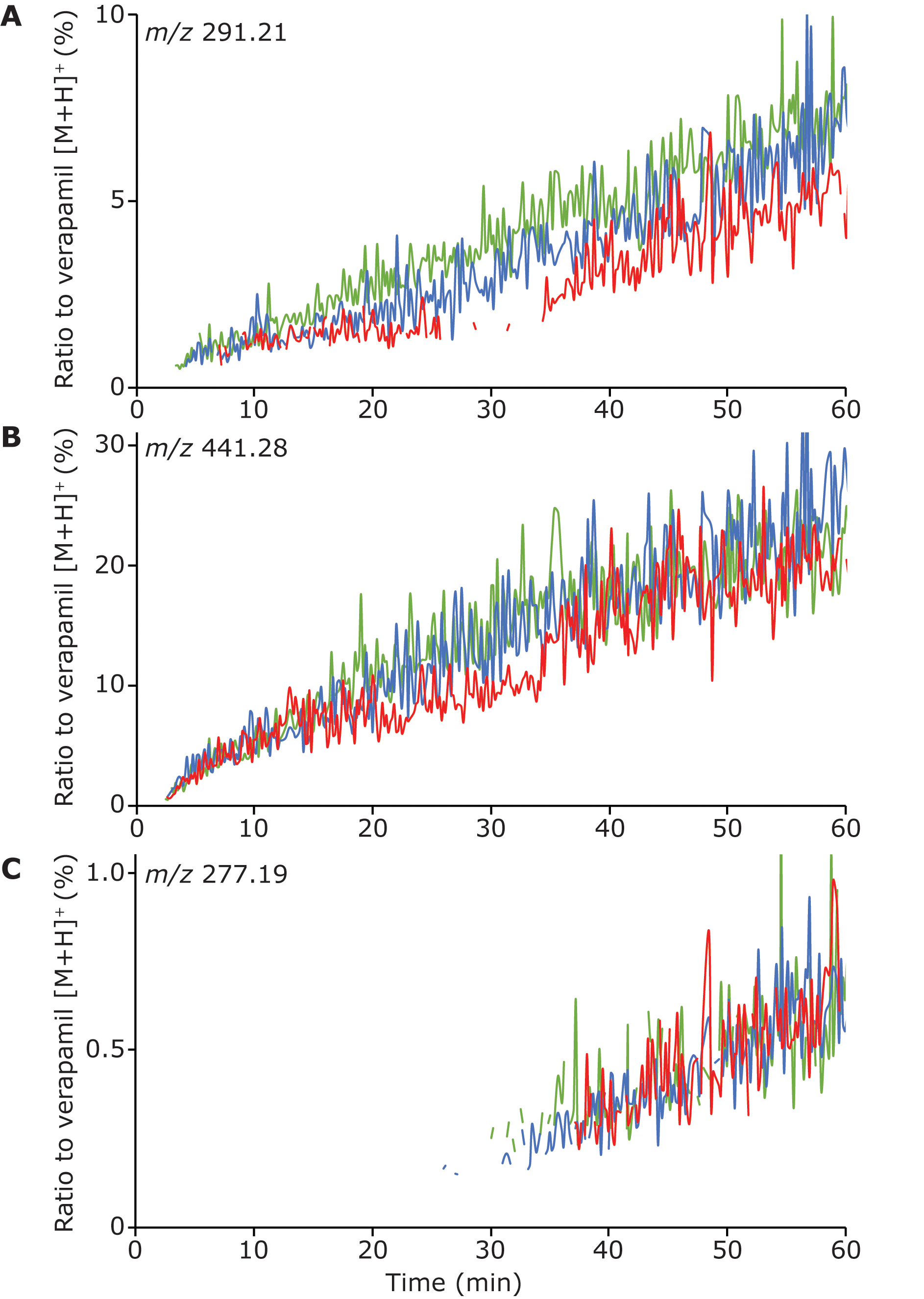


Figure S14. Individual data for oxidation products generated by time-resolved TiO_2_ photocatalysis LAESI-MS as presented in Figure 3

. Each signal is normalized to verapamil [M+H]^+^. **A** shows data for *m/z* 291.21 (N-dealkylation). **B** presents data for *m/z* 441.28 (demethylation) and **C** depicts data for *m/z* 277.19 (both the N-dealkylation and demethylation).

References

1. Walles, M., Thum, T., Levsen, K., Borlak, J.: Verapamil: new insight into the molecular mechanism of drug oxidation in the human heart. J. Chromatogr. A **970**, 117-130 (2002)

2. Ruokolainen, M., Miikkulainen, V., Ritala, M., Sikanen, T., Kotiaho, T., Kostiainen, R.: TiO_2_ photocatalysis–DESI-MS rotating array platform for high-throughput investigation of oxidation reactions. Anal. Chem. **89**, 11214-11218 (2017)

3. Calza, P., Pazzi, M., Medana, C., Baiocchi, C., Pelizzetti, E.: The photocatalytic process as a tool to identify metabolitic products formed from dopant substances: the case of buspirone. J. Pharm. Biomed. Anal. **35**, 9-19 (2004)

4. Zhu, M., Zhao, W., Jimenez, H., Zhang, D., Yeola, S., Dai, R., Vachharajani, N., Mitroka, J.: Cytochrome P450 3A-mediated metabolism of buspirone in human liver microsomes. Drug Metab. Dispos. **33**, 500-507 (2005)

5. Thevis, M., Beuck, S., Höppner, S., Thomas, A., Held, J., Schäfer, M., Oomens, J., Schänzer, W.: Structure elucidation of the diagnostic product ion at *m/z* 97 derived from androst-4-en-3-one-based steroids by ESI-CID and IRMPD spectroscopy. J. Am. Soc. Mass. Spectrom. **23**, 537-546 (2012)

6. Williams, T. M., Kind, A. J., Houghton, E., Hill, D. W.: Electrospray collision‐induced dissociation of testosterone and testosterone hydroxy analogs. J. Mass Spectrom. **34**, 206-216 (1999)

7. Thevis, M., Thomas, A., Fußhöller, G., Beuck, S., Geyer, H., Schänzer, W.: Mass spectrometric characterization of urinary metabolites of the selective androgen receptor modulator andarine (S‐4) for routine doping control purposes. Rapid Commun. Mass Spectrom. **24**, 2245-2254 (2010)
